# Operational Safety Assessment of Turbo Generators with Wavelet Rényi Entropy from Sensor-Dependent Vibration Signals

**DOI:** 10.3390/s150408898

**Published:** 2015-04-16

**Authors:** Xiaoli Zhang, Baojian Wang, Xuefeng Chen

**Affiliations:** 1 Key Laboratory of Road Construction Technology and Equipment, Ministry of Education, Chang’an University, Xi’an 710064, China; 2 State Key Laboratory for Manufacturing and Systems Engineering, Xi’an Jiaotong University, Xi’an 710049, PR China; E-Mails: wangbaojian@mail.xjtu.edu.cn (B.W.); chenxf@mail.xjtu.edu.cn (X.C.)

**Keywords:** operational safety assessment, turbo generator, sensor-dependent, vibration signal, wavelet Rényi entropy, second generation wavelet package

## Abstract

With the rapid development of sensor technology, various professional sensors are installed on modern machinery to monitor operational processes and assure operational safety, which play an important role in industry and society. In this work a new operational safety assessment approach with wavelet Rényi entropy utilizing sensor-dependent vibration signals is proposed. On the basis of a professional sensor and the corresponding system, sensor-dependent vibration signals are acquired and analyzed by a second generation wavelet package, which reflects time-varying operational characteristic of individual machinery. Derived from the sensor-dependent signals’ wavelet energy distribution over the observed signal frequency range, wavelet Rényi entropy is defined to compute the operational uncertainty of a turbo generator, which is then associated with its operational safety degree. The proposed method is applied in a 50 MW turbo generator, whereupon it is proved to be reasonable and effective for operation and maintenance.

## 1. Introduction

Turbo generators are a key part of power systems which have found increasing service in the power industry throughout the world. They can produce a great amount of electrical energy depending on their size and weight. They usually require regular upkeep and scheduled maintenance. With a detailed, long term maintenance plan in place, utilities can ensure that their facilities will safely deliver as much reliable power to the grid as possible. The criteria for turbo generator is high reliability, high performance, with many starts and flexible operation throughout the service life [1]. In addition, modern turbo generators are built to last between 30 and 40 years. With aging generator units and mechanical components, safety assessment is one of the most important and imperative indicators for a plant to prevent failures.

Safety refers to the ability of a system or component to perform its required function under stated conditions for a specified period of time without accidents, which is very important for industrial enterprises to protect running reliability against damage, faults, failures and economic losses. Therefore, safety is studied worldwide by many researchers and engineers. Matteson proposed a dynamic multi-criteria optimization framework for sustainability and reliability assessments of power systems [2]. Lo Prete proposed a framework to assess and quantify the sustainability and reliability of different power production scenarios [3]. Moharil *et al*. analyzed the generator system reliability with wind energy penetration in the conventional grid [4]. Since turbo generator faults have a significant impact on safety, Whyatt *et al*. identified failure modes experienced by turbo generators and described their reliability [5]. Tsvetkov *et al*. presented a mathematical model for analysis of generator reliability, including development of defects [6].

Generally speaking, traditional approaches entail collecting sufficient failure samples to estimate the general probability of the system or component failures and the distribution of the time-to-failure. It is usually difficult to use probability and statistics for turbo generator safety analysis due to the lack of failure samples and time-to-failure data. The failure rate of a generator includes all the failures which cause the generator to shut down and also depends on the maintenance and operating policy of utilities. In fact, turbo generators are usually set on different operating parameters and conditions (e.g., temperatures, vibration, load, stress). The variations of the operating parameters can affect operational safety whenever a single parameter or condition is out of limit and failures can also be caused by the interaction of operating parameters. It has been realized from the real-time operation that a component will experience more failures during heavy loading conditions than during light loading conditions, which means that the failure rate of a component in real-time operation is not constant and varies with operating parameters [7]. Depending on the operating parameters and conditions, the constitutive components of a turbo generator will go through a series of degradation states evolving from functioning to failure. Therefore, there is a great demand of ways of assessing the operational safety of turbo generators with time-varying operational parameters and conditions during their whole life span, which is beneficial for implementing optimal condition-based maintenance schedules with low failure risk.

When condition monitoring is performed during plant operational transients, the intrinsically dynamic behavior of the monitored time-varying signals should be taken into account [8,9,10]. Monitoring the condition of a component is typically based on several sensors that estimate the values of some measurable parameters (signals) and triggering a fault alarm when the measured signal is out of limit. To this purpose, Baraldi *et al.* proposed approaches based on the development of several reconstruction models and the signals were preprocessed by means of Haar wavelet transforms for a gas turbine during start-up transients [8]. Lu *et al.* proposed a simplified on-board model with sensor fault diagnostic logic for turbo-shaft engines [11]. Li *et al.* established a hybrid model for hydraulic turbine-generator units based on nonlinear vibration [12]. Hua *et al.* proposed a novel performance degradation estimation method based on an adaptive failure threshold and degradation signal data series [13]. The above operational safety diagnosis and assessment methods have mainly utilized dynamic monitored information, so how to process the monitored information and associate it with operational safety is very essential.

Information entropy is an effective indicator to measure a system’s degree of uncertainty. On the basis of the information entropy theory, the most uncertain probability distribution (such as the equal probability distribution) has the largest entropy, and the most certain probability distribution has the smallest entropy. On this basis, the use of information entropy is widespread in engineering applications. Different types of information entropy have been defined in accordance with their own usage, such as topological entropy of a given interval map [14], spatial entropy of pixels [15], weighted multiscale permutation entropy of nonlinear time series [16], Shannon differential entropy for distributions [17], min-and max-entropies [18], collision entropy [19], permutation entropy [20], time entropy [21], multiscale entropy [22], wavelet entropy [23] and so on.

Entropy is well used in machinery fault diagnosis. Sawalhi *et al.* used minimum entropy and spectral kurtosis for fault detection in rolling element bearings [24]. Tafreshi *et al.* proposed a machinery fault diagnosis method utilizing entropy measure and energy map [25]. He *et al.* approximated entropy as a nonlinear feature parameter for fault diagnosis of rotating machinery [26]. Wu *et al.* proposed a bearing fault diagnosis method based on multiscale permutation entropy and support vector machine [27].

In the branch of information entropy, Rényi entropy was introduced by Alfréd Rényi in 1960 [28], It is known as a parameterized family of uncertainty measures. It is noteworthy that the classical Shannon entropy [29,30] is a special case of Rényi entropy when the order *α* of Rényi entropy is equal to one. Similarly, other entropy measures that have appeared in various literatures are also special cases of Rényi’s entropy [31]. Besides being of theoretical interest as a unification of several distinct entropy measures, Rényi entropy has found various applications in statistics and probability [32], pattern recognition [33], quantum chemistry [34], biomedicine [35], *etc*.

Therefore, a new method of operational safety evaluation based on wavelet Rényi entropy from sensor-dependent vibration signals is proposed. Firstly, the sensor-dependent vibration signals reflecting the time-varying characteristic of an individual turbo generator are acquired by professional sensors and then analyzed by the second generation wavelet package since the wavelet transform excels in analyzing unsteady signals in both the time domain and frequency domain. Derived from the sensor-dependent signals’ wavelet energy distribution over the observed frequency range, wavelet Rényi entropy is defined to compute the operational uncertainty, which is then transformed to an operational safety degree. Finally, the proposed method is applied in a 50 MW turbo generator to validate the effectiveness of the proposed method for operation and maintenance.

The organization of the paper is as follows: the basic theory of probability and Rényi entropy is briefly reviewed in Section 2. The proposed operational safety assessment method with wavelet Rényi entropy from sensor-dependent vibration signals is explained in Section 3. A case study of a 50 MW turbo generator is conducted in Section 4. General conclusions are drawn in Section 5.

## 2. Theory Background

### 2.1. Probability Space and Random Variable

As usual, a finite probability space is given by a non-empty finite set Ω and a probability function P:Ω→[0,1] with $\underset{\omega \in \text{Ω}}{\sum }P\left(\omega \right)=1$, taking it as understood that the σ-algebra is given by the power set of Ω [32]. For a random variable X:Ω→χ, where its range χ is assumed to be finite. The distribution of X is denoted as $P_{X}:\chi \to [0,1]$, *i.e.*, $P_{X}\left(x\right)=P(X=x)$, where X=x is a shorthand for the event {ω∈Ω|X(ω)=x}. The standard notation for intervals in ℝ, e.g., [0,1] and [1,∞] are denoted the respective intervals [0,1]={*r*∈ℝ*|*0≤ *r*≪1}and [1,∞] ={*r* ∈ℝ*|*1< *r* }.

### 2.2. Rényi Entropy

Rényi entropy unifies all the distinct entropy measures. For a parameter $\text{α}\in \left[0,1\right)\cup^{\text{​}}(1,\infty )$ and a random variable X, the Rényi entropy of X is defined as:

(1) $$H_{\alpha }(X)=\frac{1}{1-\alpha }\log\sum_{x}^{\;}P_{X}{\left(x\right)}^{\alpha }$$

where the sum is over all $x\in supp(P_{X})$.

It is well known and not hard to verify that this definition of $H_{\alpha }\;$ is consistent with the respective definitions of *H*_0_ and *H*_2_ and ${\text{ lim}}_{\alpha \to 1}\;H_{\alpha }(X)\;=\;H(X)\;$ and ${\text{ lim}}_{\alpha \to \infty }\;H_{\alpha }(X)=H_{\infty }(X)$. Furthermore, it is known that the Rényi entropy is decreasing in *α*, *i.e.*, $H_{\beta }(X)\leq H_{\alpha }(X)\;$ for  0≤ α≤β≤∞.

It will be convenient to re-write $H_{\alpha }(X)\;$ for $\text{ α}\in \left[0,1\right)\cup^{\text{​}}(1,\infty )$ as $H_{\alpha }\left(X\right)=-{\text{log Ren}}_{\alpha }(X)$ with:

(2) Renα(X)=(∑x PX(x)α)1α−1=‖PX‖ααα−1

where ${\left\|P_{X}\right\|}_{\alpha }$ is the *α*-norm of $\;P_{X}:X\to [0,\;1]\subset \mathbb{R}$, but we keep in mind that it actually is a norm only in case *α* ≥ 1. We call Ren*_α_*(*X*) the Rényi probability (of order *α*) of *X*. For completeness, we also define Ren_0_(*X*) = |supp(*P_X_* )|^−1^, and Ren_1_(*X*) = 2^−*H*(*X*)^, which is consistent with taking the limits. It is worthy to note that the parameter α is set as 0.9 in the following experiment.

## 3. Operational Safety Assessment with Wavelet Rényi Entropy from Sensor-Dependent Vibration Signals

### 3.1. Second Generation Wavelet Package Transform (SGWP)

The second generation wavelet inherits the good multi-resolution ratio characteristics and the time–frequency localization properties of the first generation wavelet transform, which is a more effective and faster implementation of the wavelet transform and has the advantages of simple construction, small amount of calculations and in-place calculation [36]. It has been proved by Daubechies that arbitrary wavelet transforms can be implemented by using a lifting scheme [37]. The second generation wavelet package transform belongs to the lifting scheme based on wavelet packet transform, which contains the forward transform (decomposition) and inverse transform (reconstruction). The inverse transform can be realized by running the forward transform backwards. The detailed procedure is explained as follows:

1. Decomposition

The forward transform of a second generation wavelet package for signal decomposition contains three steps: split, prediction and update.

*Split*: Supposing there is an original signal S={x(k),k∈Z}, the original signal can be divided into an even series $s_{e}=\{s_{e}(k),k\in Z\}$ and an odd series $s_{o}=\{s_{o}\left(k\right),k\in Z\}$:

(3) $$s_{e}(k)=x(2k),\;k\in Z$$

(4) $$s_{o}(k)=x(2k+1),\;k\in Z$$

The reason for splitting an original signal into two series is that adjacent samples are much more correlated than those far from each other. Therefore, the odd and even series are highly correlated.

**Figure 1:**
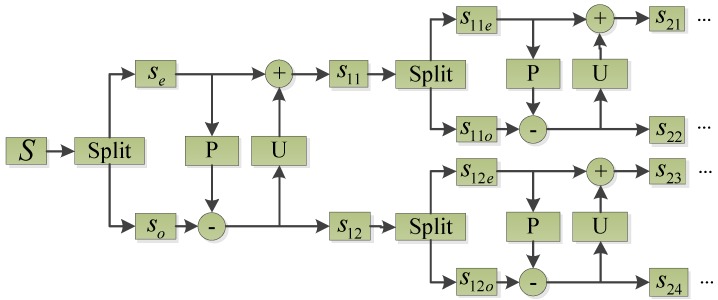
Illustration of the forward transform of second generation wavelet package.

*Prediction and update*: Several samples of even series can be used to predict a certain sample in the odd series, and the prediction difference is defined as a detail signal. The even series can be updated using the obtained detail signal and the modified even series is defined as an approximation signal:

(5) $$s_{l1}=s_{(l-1)1o}-P(s_{(l-1)1e})$$

(6) $$s_{l2}=s_{(l-1)1e}+U(s_{l1})$$

(7) $$s_{l(2^{l}-1)}=s_{(l-1)2^{l-1}o}-P(s_{(l-1)2^{l-1}e})$$

(8) $$s_{l2^{l}}=s_{(l-1)2^{l-1}e}+U(s_{l(2^{l}-1)})$$

where $s_{l1},s_{l2},\cdots ,s_{l2^{l}}$ are the decomposed signals, in each frequency band respectively, after $l^{\text{th}}$ decomposition; $s_{(l-1)1o},\cdots ,s_{(l-1)2^{l-1}o}$ are odd series respectively after the ${\left(l-1\right)}_{\text{th}}$ decomposition; $s_{(l-1)1e},\cdots ,s_{(l-1)2^{l-1}e}$ are even series respectively after the ${\left(l-1\right)}_{\text{th}}$ decomposition; P is defined as N point predictor whose prediction coefficients are $p_{1},p_{2},\cdots ,p_{N}$ and N is predictor order. U is denoted as $\tilde{N}$ point updater with update coefficients $u_{1},u_{2},\cdots ,u_{\tilde{N}}$ and $\tilde{N}$ is updater order. The coefficients $p_{1},p_{2},\cdots ,p_{N}$ and $u_{1},u_{2},\cdots ,u_{\tilde{N}}$ can be attained based on [37,38]. The forward transform of second generation wavelet package transform is illustrated in Figure 1.

2. Reconstruction

The inverse transform for signal reconstruction can be derived from the forward transform by running the lifting scheme as illustrated in Figure 1 backwards. The signal in one frequency band after decomposition is set to be reconstructed, and the others are set as zero. The signal reconstruction of second generation wavelet package transform for an appointed frequency band is carried out as follows:

(9) $$s_{(l-1)2^{l-1}e}=s_{l2^{l}}-U(s_{l(2^{l}-1)})$$

(10) $$s_{(l-1)2^{l-1}o}=s_{l(2^{l}-1)}+P(s_{(l-1)2^{l-1}e})$$

(11) $$s_{(l-1)2^{l-1}}(2k)=s_{(l-1)2^{l-1}e}(k),\;k\in Z$$

(12) $$s_{(l-1)2^{l-1}}(2k+1)=s_{(l-1)2^{l-1}o}(k),\;k\in Z$$

(13) $$s_{(l-1)1e}=s_{l2}-U(s_{l1})$$

(14) $$s_{(l-1)1o}=s_{l1}+P(s_{(l-1)1e})$$

(15) $$s_{(l-1)1}(2k)=s_{(l-1)1e}(k),\;k\in Z$$

(16) $$s_{(l-1)1}(2k+1)=s_{(l-1)1o}(k),\;k\in Z$$

where $s_{(l-1)1}(2k)、s_{(l-1)1}(2k+1),\cdots ,s_{(l-1)2^{l}}(2k)、s_{(l-1)2^{l}}(2k+1)$ are the reconstructed signals of the appointed frequency band from the ${\left(l-1\right)}_{\text{th}}$ reconstruction of the second generation wavelet package transform.

### 3.2. Energy Distribution of Second Generation Wavelet Package Transform

Since the second generation wavelet package transform obeys the energy conservation principle due to its bi-orthogonal basis, each of the attained $2^{l}$ frequency bands has the same bandwidth and end to end after the $l_{\text{th}}$ decomposition and reconstruction. Supposing $s_{l,i}(k)$ is the reconstructed signal at the $l_{\text{th}}$ decomposition in the $l_{\text{th}}$ frequency band, its energy $E_{l,i}$ and relative energy ${\tilde{E}}_{l,i}$ are respectively defined as follows:

(17) $$E_{l,i}=\frac{1}{n-1}\sum_{k=1}^{n}{\left(s_{l,i}(k)\right)}^{2},\;i=1,2,\cdots ,2^{l},\;k=1,2,\cdots ,n,\;n\in Z$$

(18) $${\tilde{E}}_{l,i}=E_{l,i}{\left(\sum_{i=1}^{2^{l}}E_{l,i}\right)}^{-1}$$

Obviously, $\sum_{i=1}^{2^{l}}{\tilde{E}}_{l,i}=1$, the sum of total relative energy equals to one.

### 3.3. Operational Safety Degree with Wavelet Rényi Entropy from Sensor-Dependent Vibration Signals

The operational safety degree with wavelet Rényi entropy R from sensor-dependent vibration signals is defined as:

(19) $$R=1-\frac{1}{1-\alpha }\log_{2^{l}}\sum_{i=1}^{2^{l}}{\left({\tilde{E}}_{l,i}\right)}^{\alpha }$$

where the parameter α∈[0,1)∪​(1,∞).

**Figure 2:**
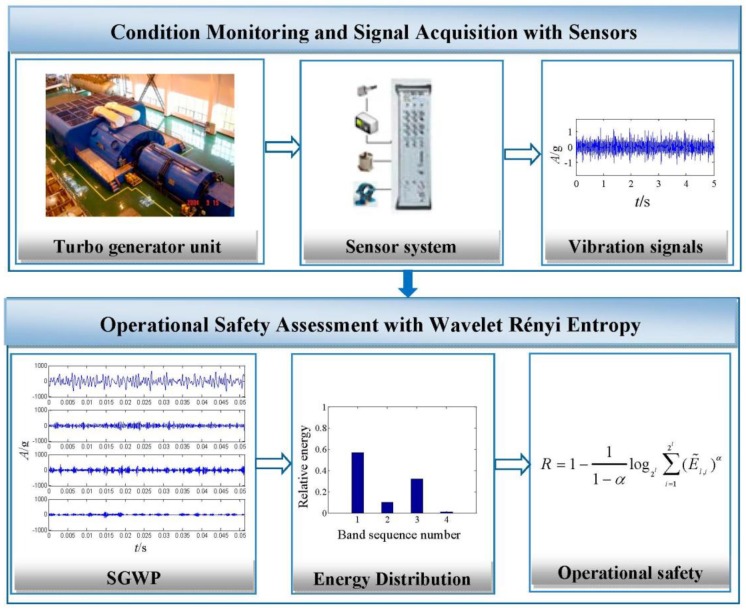
Illustration of operational safety assessment with wavelet Rényi entropy from sensor-dependent vibration signals.

An illustration of the proposed operational safety assessment method with wavelet Rényi entropy is presented in Figure 2, which mainly includes condition monitoring and signal acquisition, signal processing with second generation wavelet package transform and operational safety assessment with wavelet Rényi entropy.

## 4. Application in Operational Safety Assessment of a Turbo Generator

### 4.1. Sensor-Dependent Vibration Signal Monitoring and Acquisition

**Figure 3:**
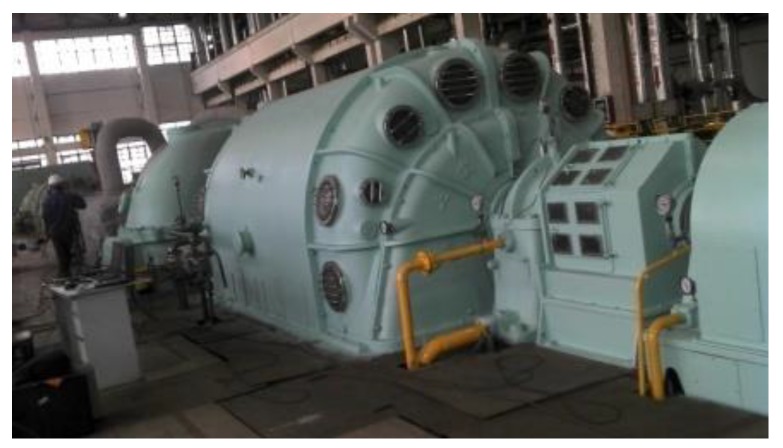
The illustration of a 50 MW turbo generator unit.

**Figure 4:**
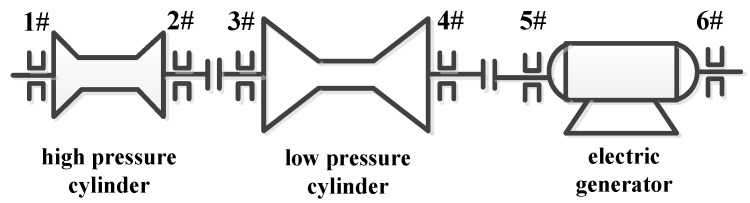
The structure diagram of the 50 MW turbo generator unit.

A comprehensive vibration monitoring study was conducted on a 50 MW turbo generator unit shown in Figure 3 to ensure its normal start-up and operation. An MDS-2 portable vibration monitoring system and professional sensors are used for vibration monitoring for the #1~#6 bearing bushings in the high pressure cylinder, low pressure cylinder and electric generator as illustrated in Figure 4. With the increased speed and load in the start-up process, all the bearings are in normal states since the peak to peak vibration in the vertical direction is less than 50 μm, except for the vibration of the #4 bearing in the low-pressure cylinder which is out of limit. Therefore, the condition monitoring emphasis is focused on the vertical vibration of the #4 bearing. In the start-up process with empty load, the peak to peak vibration in the vertical direction of #4 bearing is 24.7 μm at the speed of 740 r/min. Moreover, the peak to peak vibration in the vertical direction is increased to 63.2 μm at the speed of 3000 r/min and even to 86.0 μm at the speed of 3360 r/min.

**Figure 5:**
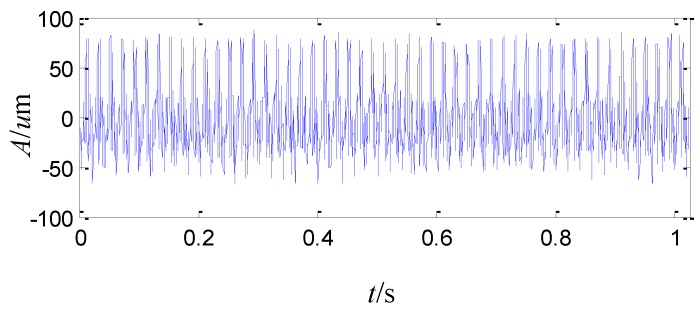
The waveform of the sensor-dependent vibration signal in time domain.

**Figure 6:**
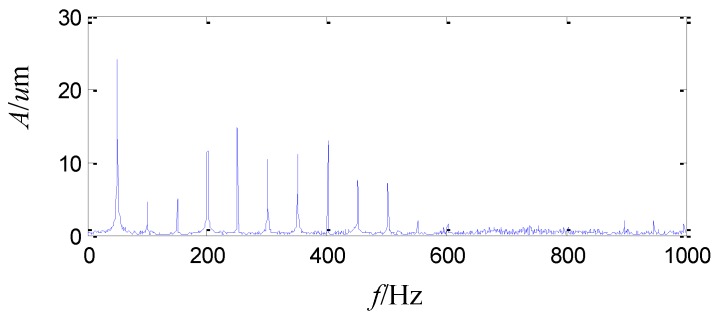
The FFT spectrum of the sensor-dependent vibration signal in frequency domain.

Afterwards, vibration monitoring is conducted in a stable speed of 3000 r/min with several given loads. The peak to peak vibration is about 74 μm with the load of 6 MW, 104 μm with the load of 16 MW, and it even increases to 132 μm with the load of 20 MW. The vibration is too severe to increase the load more, so the load is decreased to 6 MW and the peak to peak vibration is about 75~80 μm. The acquired vibration waveform is shown in Figure 5, which shows disorder and dissymmetry in the top and bottom of the vibration signal. The sampling frequency is 2 KHz. The FFT spectrum of the vibration signal is shown in Figure 6. The amplitude of the running frequency is the largest in the whole frequency range. In addition, there are some harmonic frequency components from two times the running frequency to ten times the running frequency, whose amplitudes are also large.

### 4.2. Sensor-Dependent Vibration Signal Analysis

In order to further analyze the sensor-dependent vibration signal, a second generation wavelet package is adopted to decompose the original signal to the extent of level 2, level 3 and level 4, respectively. Each frequency band has the same bandwidth end to end. Afterwards, the relative energy of the corresponding frequency band analyzed by the second generation wavelet package is computed according to Equation (18).

**Figure 7:**
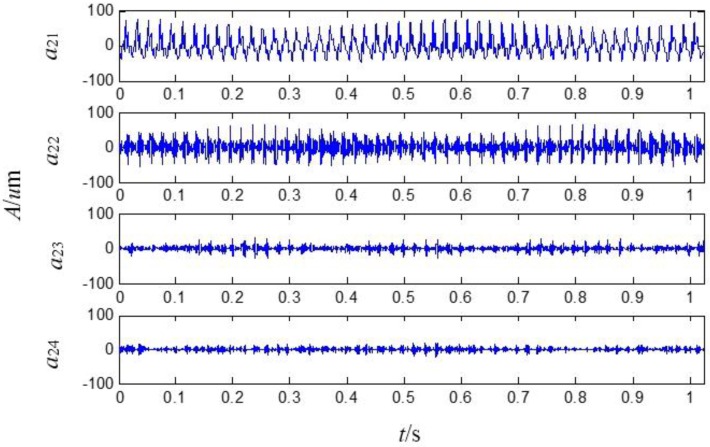
The sensor-dependent signals analyzed by the second generation wavelet package in level 2.

The four signals obtained analyzed by the second generation wavelet package in level 2 are illustrated in Figure 7, which respectively correspond to the frequency bands of 0~250 Hz, 250~500 Hz, 500~750 Hz and 750~1000 Hz.

**Figure 8:**
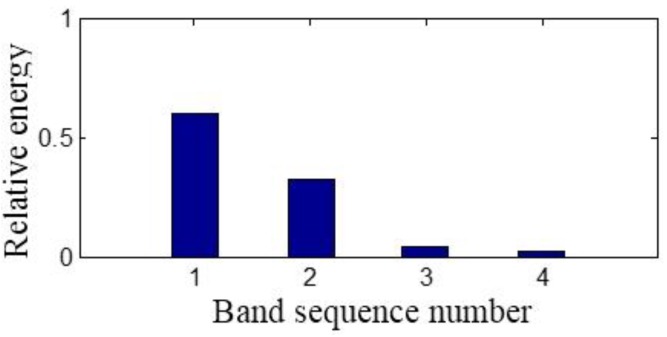
The sensor-dependent signal’s relative energy distribution in level 2.

The sensor-dependent signal’s relative energy of the corresponding frequency band is calculated according to Equation (18), which is shown in Figure 8. The relative energy of the first frequency band is the largest, while the relative energy of the second frequency band is larger than that of the last two frequency bands.

**Figure 9:**
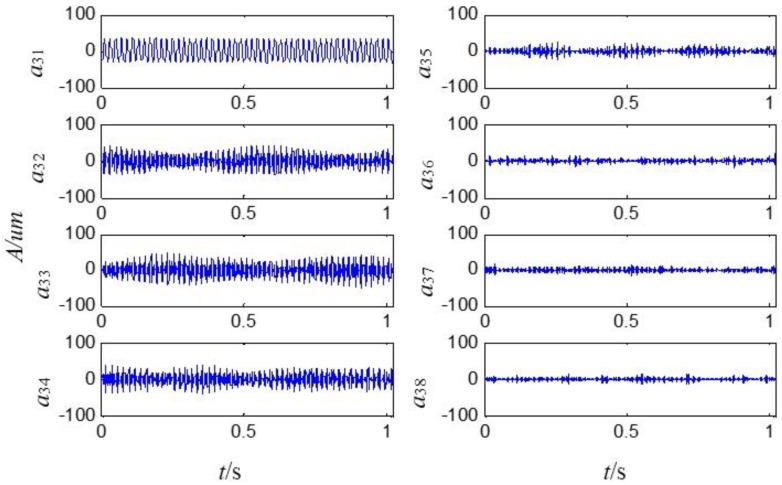
The sensor-dependent signals analyzed by second generation wavelet package in level 3.

**Figure 10:**
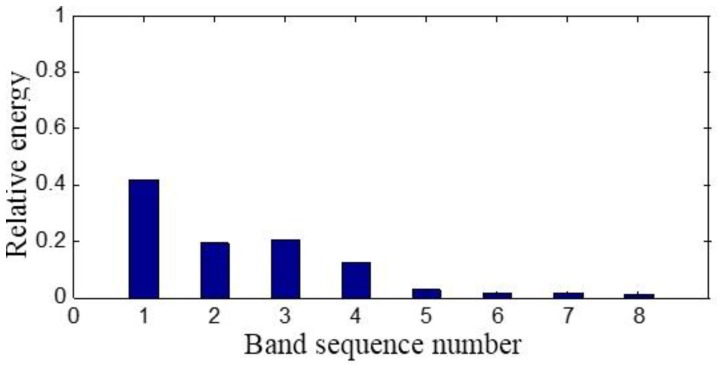
The sensor-dependent signal’s relative energy distribution in level 3.

On the basis of the level 2 study, the original signal is further decomposed to the extent of level 3 as shown in Figure 9. The eight signals obtained correspond to the frequency bands of 0~125 Hz, 125~250 Hz, 250~375 Hz, 375~500 Hz, 500~625 Hz, 625~750 Hz, 750~875 Hz and 875~1000 Hz. The relative energy is distributed in the eight frequency bands as shown in Figure 10. The energy of the first four frequency bands is much larger than that of the rest.

**Figure 11:**
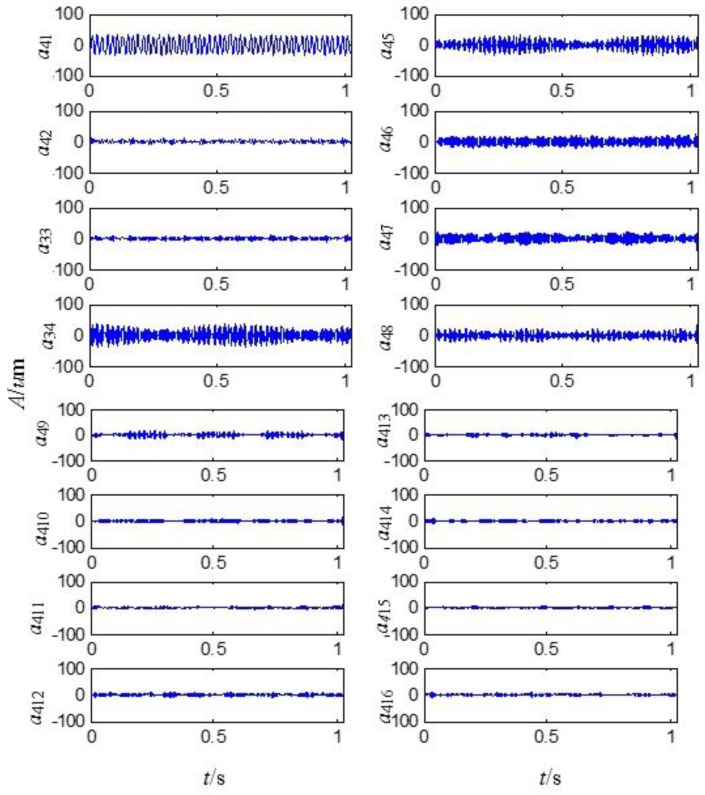
Signals analyzed by second generation wavelet package in level 4.

**Figure 12:**
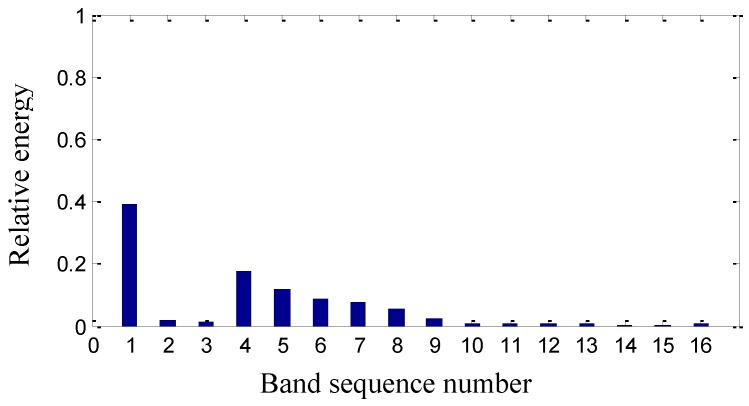
Wavelet package energy distribution in level 4.

On the basis of the level 3 study, the original signal is further decomposed to the extent of level 4 as shown in Figure 11. The obtained sixteen signals correspond to the frequency bands of 0~62.5 Hz, 62.5~125 Hz, 125~187.5 Hz, 187.5~250 Hz, 250~312.5 Hz, 312.5~375 Hz, 375~437.5 Hz, 437.5~500 Hz, 500~562.5 Hz, 562.5~625 Hz, 625~687.5 Hz, 687.5~750 Hz, 750~812.5 Hz, 812.5~875 Hz, 875~937.5 Hz and 937.5~1000 Hz. The signal’s relative energy of each frequency band is computed as shown in Figure 12.

The relative energy of the first band is much larger than that of the other frequency bands. Except for the first largest frequency bands, the energy of the fourth to the ninth frequency band is larger than that of the rest. It is inferred that there are some fault information contained in the fourth to the ninth frequency band.

### 4.3. Operational Safety Assessment with Wavelet Rényi Entropy from Sensor-Dependent Vibration Signals

The operational safety degree is calculated according to Equation (19) from level *l* = 2 to level *l* = 4, respectively. From Table 1, it is seen that the current operational performance of the turbo generator is not suitable, since all of the calculated safety degrees from level *l* = 2 to level *l* = 4 are under 0.4, the lowest of which is 0.2467 in level *l* = 3. Since mechanical faults and unhealthy parameters can make machinery operational condition become uncertain, the probability distribution of the monitored vibration signal become uncertain and thus induces a much lower operational safety.

**Table 1:** The operational safety degree before maintenance from sensor-dependent vibration signals.

Decomposition Level	*l* = 2	*l* = 3	*l* = 4
Operational safety degree	0.3363	0.2467	0.2812

### 4.4. Fault Diagnosis

As the above analysis, the amplitude of the running frequency is the largest among the whole frequency range and some harmonic frequency components from two times the running frequency to ten times the running frequency are also large in Figure 6. Signals analyzed by the second generation wavelet package and wavelet package energy distributions in level 2, level 3 and level 4 exhibit non-stationary, nonlinear and colored noise characteristics.

Considering the start-up process with no load and loading operation conditions, the vertical vibrations of the #3 and #5 bearings which are adjacent to the #4 bearing are not high (under 20 m). Different from the #3 and #5 bearings, the vibration of the #4 bearing increases with increased speed and load. It is concluded that the vibration is not caused by imbalance and misalignment for the reason that vibrations would be out of limits in multiple bearing position if an imbalance or misalignment fault occurs. Therefore, the problem is focused on the #4 bearing itself. It is inferred that the monitored non-stationary and nonlinear components in the vibration signal of the #4 bearing may be caused by mechanical looseness and local friction, so the bearing force and support status of the sizing block and bearing lodgement must be checked.

With the above analysis, the turbo generator unit is stopped and overhauled. The preload of the #4 bearing bushing is about 0.11 mm, which is far from the requirement of 0.25 mm. The gaps of the left and right sizing block are checked by a filler gauge. The 0.05 mm filler gauge can be filled into 30 mm of the left sizing block and 25 mm of the right sizing block. The gap in the bottom of the #4 bearing bushing is also far away from the obligate gap of 0.05 mm. Therefore, the gaps of the 4# bearing bush are re-corrected and the preload is added to the requirement of 0.25 mm.

**Figure 13:**
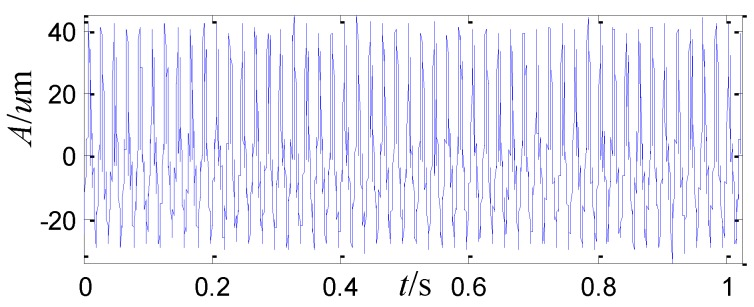
The waveform of the sensor-dependent vibration signal in time domain after maintenance.

**Figure 14:**
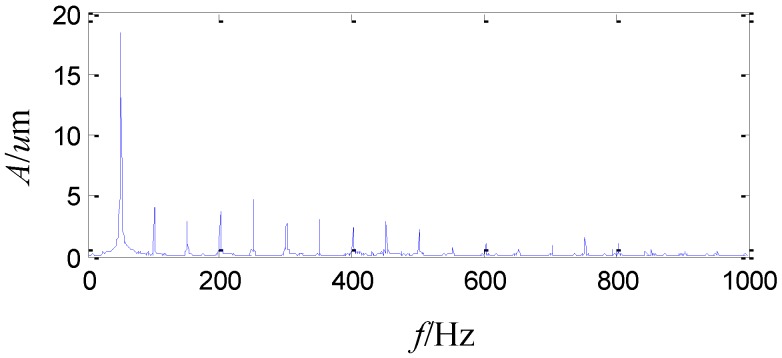
The FFT spectrum of the sensor-dependent vibration signal after maintenance.

After maintenance, the turbo generator unit is operated again. The sensor-dependent vibration signal is decreased in the start-up process with increasing speed. The peak to peak vibration in the vertical direction in the #4 bearing bushing is about 40~55 μm with a load of 45 MW, which is much better than before. In order to assess the operational safety of the turbo generator unit after maintenance, vibration monitoring via sensors is conducted in a stable speed of 3000 r/min with a load of 6 MW, which is as the same as the case before maintenance. The waveform of the acquired vibration signal shown in Figure 13 shows some differences compared with the vibration signal before maintenance in Figure 5, such as the symmetry between the top and bottom of the vibration signal is much better than before and the peak to peak vibration is about 45 μm, which falls in the permissible range. The FFT spectrum of the vibration signal is shown in Figure 14, which is different from the spectrum before maintenance shown in Figure 6. The amplitudes of the harmonic frequency components from two times the running frequency to ten times the running frequency are decreased. The second generation wavelet package is adopted to analyze the acquired vibration signal on level 2, level 3 and level 4, respectively. Afterwards, the relative energy of the corresponding frequency band analyzed by the second generation wavelet package is computed according to the Equation (18).

**Figure 15:**
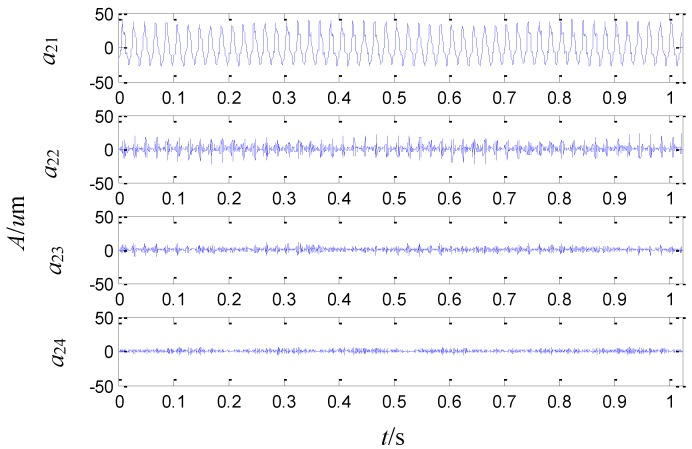
Signal analysis by the second generation wavelet package in level 2 after maintenance.

**Figure 16:**
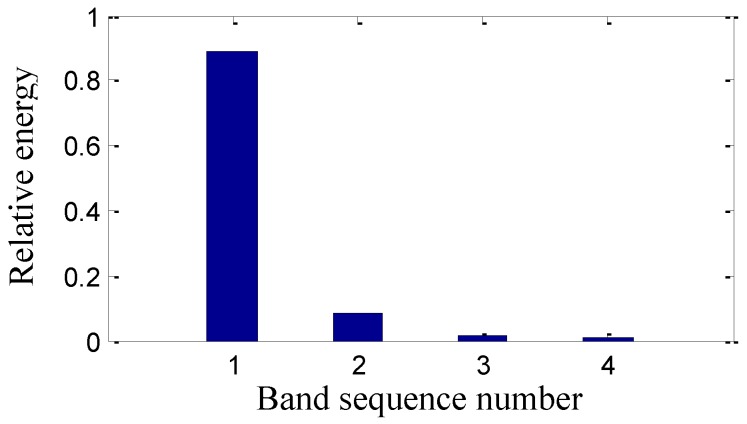
The sensor-dependent signal’s relative energy distribution after maintenance.

The four obtained signals analyzed by the second generation wavelet package in level 2 are illustrated in Figure 15. The signals’ relative energy after maintenance is concentrated in the first frequency band shown in Figure 16 and the relative energy of the last three frequency bands is very little, which is quite different from Figure 8. It is shown that the relative energy in the second frequency band of Figure 8 before maintenance is generated by the machinery fault information of the gaps in the #4 bearing bushing and the whole energy is decentralized in frequency bands.

**Figure 17:**
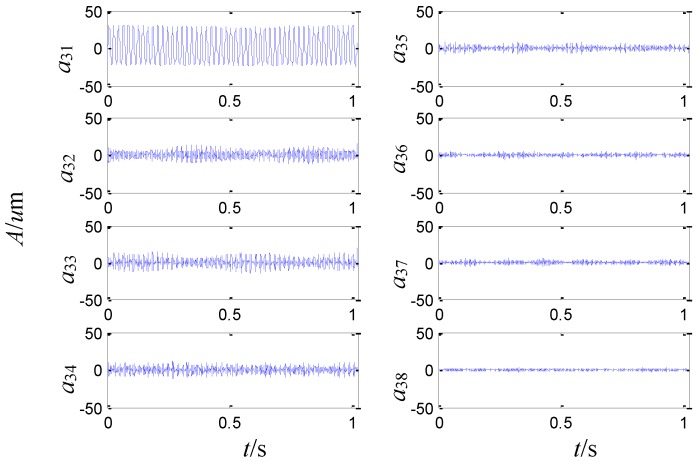
Signal analysis by the second generation wavelet package in level 3 after maintenance.

**Figure 18:**
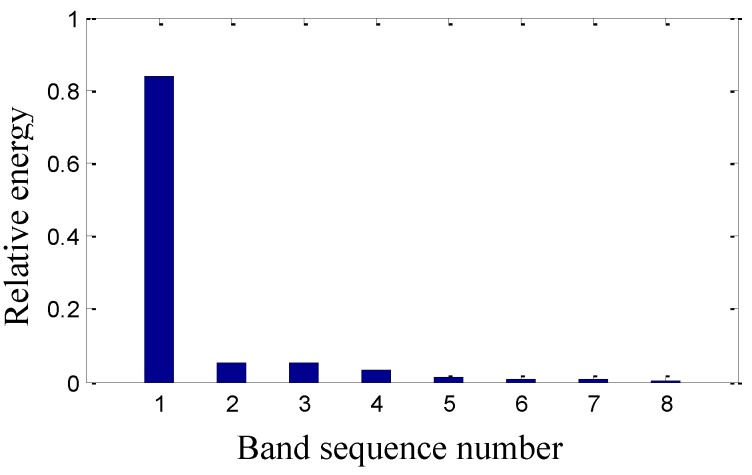
The sensor-dependent signal’s relative energy distribution in level 3 after maintenance.

The sensor-dependent vibration signal is processed to the extent of level 3, which is shown in Figure 17. The signals’ relative energy distribution shown in Figure 18 is also different from that in Figure 10 before maintenance, such as the relative energy of the last seven frequency bands is very small in Figure 18.

**Figure 19:**
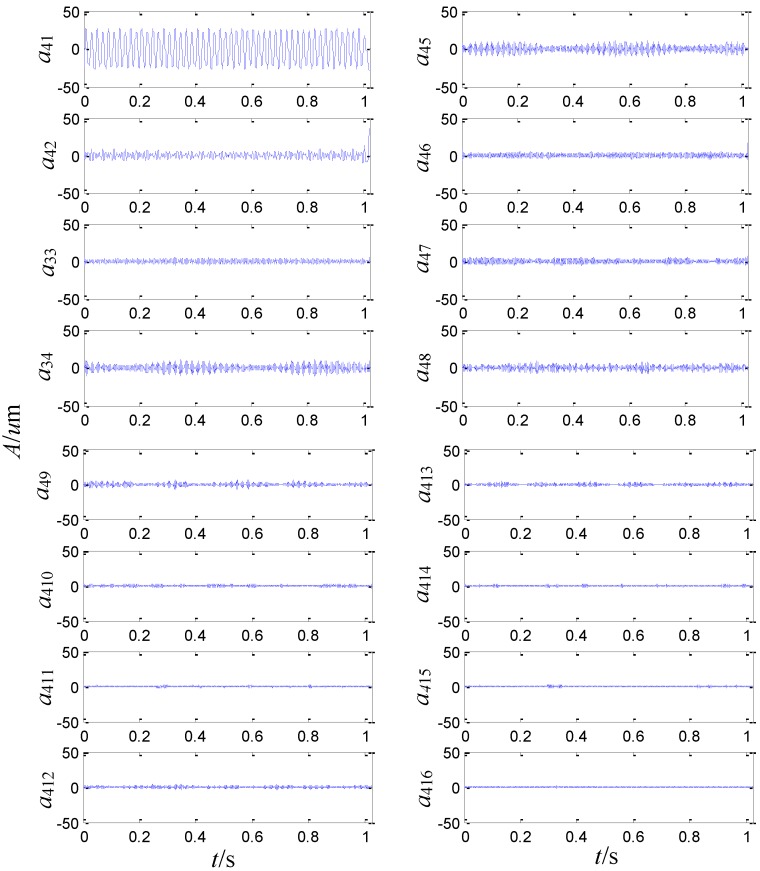
Signals analysis by second generation wavelet package in level 4 after maintenance.

It is inferred that the relative energy from the second frequency band to the fourth frequency band in Figure 10 is caused by the machinery’s fault information concerning the gaps in the #4 bearing bushing. The sensor-dependent vibration signal is finally processed to the extent of level 4, which is shown in Figure 19.

**Figure 20:**
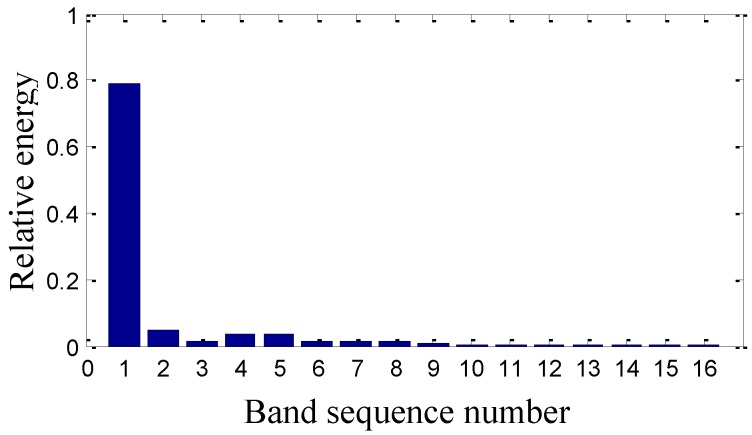
The sensor-dependent signal’s relative energy distribution in level 4 after maintenance.

As shown in Figure 20, the signal’s relative energy distribution after maintenance is also different from that before maintenance in Figure 12, such as the relative energy in the rest frequency bands is very little except the first one in Figure 20.

It is inferred that the relative energy from the fourth frequency band to the ninth frequency band in Figure 12 before maintenance is caused by the machinery fault information of the gaps in the #4 bearing bushing. It is concluded that machinery fault information can spoil the energy convergence of the second generation wavelet package and thus induce the dispersion of the wavelet energy distribution. Therefore, it is verified that second wavelet package transform can process the vibration signals in different frequency bands to effectively reveal the machinery operation conditions.

To summarize, it is diagnosed that the extensive vibration is caused by the looseness of the #4 bearing, poor support and tension force shortage. The vibrations added with increased speed and load, which have the characteristics of non-stationarity, nonlinear properties and contain colored noise because of the friction caused by looseness faults.

### 4.5. Operational Safety Assessment after Maintenance

The operational safety degree of the turbo generator after maintenance is calculated according to Equation (19) from level *l* = 2 to level *l* = 4, respectively, in Table 2. It is seen that the operational safety degree is improved after maintenance.

**Table 2:** The operational safety degree before maintenance from sensor-dependent vibration signals after maintenance.

Decomposition Level	*l* = 2	*l* = 3	*l* = 4
Operational safety degree	0.8627	0.8278	0.8060

### 4.6. Discussion on the Operational Safety Assessment Influenced by Decomposition Level l of the Second Generation Wavelet Package Transform

From Table 1 and Table 2, it is seen that the calculated operational safety degree is closely related to the decomposition level *l* of the second generation wavelet package transform. When the decomposition level *l* increases, the number of frequency bands is increased and the initially concentrated signal’s wavelet energy is scattered with the increased frequency bands. Each frequency band occupies a certain energy and the signal’s wavelet energy distribution becomes more uncertain. In Table 2, the computed operational safety is monotonously decreased with increasing level from level *l* = 2 to level *l* = 4, but in Table 1 before maintenance, the monotone decreasing rule is not obeyed since the machinery fault information makes the signal’s wavelet energy distribution decentralized. Therefore, the operational safety should be computed at an appropriate level *l*. Level *l* = 3 is suggested as a very suitable level since it is a middle level between level *l* = 2 to level *l* = 4, which is not too large or too small.

### 4.7. Discussion of the Operational Safety before and after Maintenance

**Figure 21:**
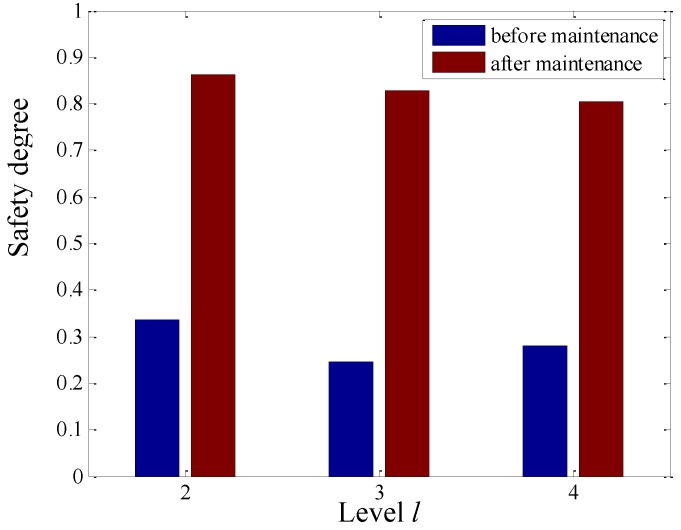
Contrasting operational safety degree before and after maintenance for different levels.

When turbo generator is degrading or unhealthy, its operational conditions will become more and more uncertain. Therefore, the wavelet Rényi entropy of the sensor-dependent vibration signal will increase and the operational safety will correspondingly decrease. The operational safety before and after maintenance is contrasted in Figure 21, where it is seen that the operational safety after maintenance is greater than before at different levels *l* of the second generation wavelet package transform. From Table 1, it is shown that all of the calculated operational safety degrees from level *l* = 2 to level *l* = 4 are under 0.4, the lowest of which is 0.2467 in level *l* = 3. The computed operational safety is very low, which illustrates that the current operational condition is seriously poor and maintenance is needed. While overhauling, it is found that the preload of the #4 bearing bushing is below standard and there are some gaps in the left and right sizing block of its bearing bushing, which induced a disorder phenomenon in the vibration signals acquired by the sensors.

After maintenance, all of the calculated operational safety degrees from level *l* = 2 to level *l* = 4 are over 0.8 in Table 2, the highest of which is 0.8627 in level *l* = 2. It is seen that maintenance in time can increase the operational safety and avoid the occurrence of accidents, so it is concluded that the proposed operational safety assessment with wavelet Rényi entropy from condition-dependent signals can provide a basis for condition-based maintenance of turbo generators.

### 4.8. Operational Safety Assessment with Wavelet Rényi Entropy in Comparison with Wavelet Entropy

In order to illustrate the effectiveness of the proposed operational safety assessment method with wavelet Rényi entropy, the operational safety degree with wavelet entropy R from sensor-dependent vibration signals is defined as:

(20) $$\tilde{R}=1-\sum_{i=1}^{2^{l}}{\tilde{E}}_{l,i}\log_{2^{l}}\;{\tilde{E}}_{l,i}$$

which is in contrast with the wavelet Rényi entropy defined in Equation (19).

The operational safety degree of wavelet entropy is calculated according to Equation (20) from level *l* = 2 to level *l* = 4, respectively. The comparison results of wavelet entropy and wavelet Rényi entropy are shown in Table 3. It is seen that the operational safety degrees are more improved by the wavelet Rényi entropy since the operational safety degrees computed from the wavelet Rényi entropy are smaller than the wavelet entropy before maintenance and the operational safety degrees computed from the wavelet Rényi entropy are increased after maintenance from level *l* =2 to level *l* = 4, so the differences between before maintenance and after maintenance using the wavelet Rényi entropy are bigger than those of wavelet entropy, and are helpful to distinguish the two conditions of turbo generator—before maintenance and after maintenance.

**Table 3:** Operational safety assessment with wavelet Rényi entropy in comparison with wavelet entropy.

Method	Decomposition Level	*l* = 2	*l* = 3	*l* = 4
wavelet entropy	Before maintenance	0.3564	0.2631	0.3034
	After maintenance	0.6956	0.6663	0.6493
	Difference between before and after maintenance	0.3392	0.4032	0.3459
wavelet Rényi entropy	Before maintenance	0.3363	0.2467	0.2812
	After maintenance	0.8627	0.8278	0.8060
	Difference between before and after maintenance	0.5264	0.5811	0.5248

## 5. Conclusions

A new method of operational safety assessment based on wavelet Rényi entropy from sensor-dependent vibration signals is proposed for turbo generators, which is realized by analyzing the sensor-dependent vibration signals. The vibration signals are analyzed by means of a second generation wavelet package. Deriving from the signal’s wavelet energy distribution over the observed frequency range, the wavelet Rényi entropy is defined to compute the operational uncertainty, which is then transformed into an operational safety degree. A case study of a 50 MW turbo generator has been studied to evaluate the operational safety before maintenance and after maintenance, which achieves desirable results. The operational safety as influenced by the decomposition level *l* of the second generation wavelet package is analyzed and set at an appropriate level. Since timely maintenance can increase the operational safety and avoid the occurrence of accidents, the proposed operational safety assessment method can serve as a basis for condition-based maintenance of turbo generators.
